# Deletion of DJ-1 in rats affects protein abundance and mitochondrial function at the synapse

**DOI:** 10.1038/s41598-020-70486-0

**Published:** 2020-08-13

**Authors:** Mohannad A. Almikhlafi, Kelly L. Stauch, Lance M. Villeneuve, Phillip R. Purnell, Benjamin G. Lamberty, Howard S. Fox

**Affiliations:** 1 Department of Pharmacology and Experimental Neuroscience, College of Medicine, University of Nebraska Medical Center, Omaha, NE USA; 2 Department of Pharmacology and Toxicology, Collage of Pharmacy, Taibah University, Medina, Saudi Arabia; 3 Department of Neurological Sciences, College of Medicine, University of Nebraska Medical Center, Omaha, NE 68198 USA; 4 Present Address: Department of Neurosurgery, Collage of Medicine, University of Oklahoma, Oklahoma City, OK USA; 5 Present Address: Otolaryngology/Head and Neck Surgery, School of Medicine, West Virginia University, Morgantown, WV USA

**Keywords:** Parkinson's disease, Energy metabolism

## Abstract

DJ-1 is a multifunctional protein affecting different biological and cellular processes. In addition, DJ-1 has roles in regulating mitochondrial function. Loss-of-function mutations in DJ-1 were found to cause an autosomal recessive form of Parkinson’s disease. One of the main pathological features of PD is loss of dopamine neurons in the nigrostriatal pathway. DJ-1 knockout (KO) rats exhibit progressive nigral neurodegeneration with about 50% dopaminergic cell loss at 8 months of age. In order to assess the effects of DJ-1 deficiency on neuronal mitochondria prior to neuron loss, we performed proteomic analysis of synaptic mitochondria isolated from the striatum, the location of nigrostriatal pathway nerve terminals, of 3-month-old DJ-1 KO rats. In total, 371 mitochondrial proteins were quantified, and of these 76 were differentially expressed in DJ-1 KO rats. Proteins perturbed by the loss of DJ-1 were involved in several mitochondrial functional pathways, including the tricarboxylic acid cycle and electron transport chain. Thus, synaptic mitochondrial respiration was measured and showed a significant change due to DJ-1 deficiency. The dataset generated here highlights the role of synaptic mitochondria in PD associated with DJ-1. This study improves our understanding of DJ-1 effects in a complex tissue environment and the synaptic mitochondrial changes that accompany its loss.

## Introduction

Parkinson’s disease (PD), is a chronic neurodegenerative movement disorder that occurs in 1% of the population older than 65 years. It is characterized by a progressive loss of dopaminergic neurons in the substantia nigra pars compacta (SNpc) and their projections into the striatum, which leads to the clinical features of the disease, including rigidity, resting tremor, bradykinesia, and gait difficulty^1^. There are two types of PD, sporadic and familial genetic. Around 90% of PD cases occur in a sporadic manner; however, approximately 10% are linked to familial genetic mutations in several different protein-encoding genes, including DJ-1^2^.

DJ-1, a highly conserved 189 amino acids protein, belongs to the DJ-1/Thi/PfpI superfamily^3^. DJ-1 is expressed in an almost all cells and tissues, including the brain^4^, and at the subcellular level is found in the cytoplasm, as well as the mitochondrial intermembrane space and matrix^3^. Bonifati and his colleagues in 2003 discovered that mutations in the gene *Park7*, which encodes the protein DJ-1, are linked with an early onset form of recessive PD^5^. All known DJ-1 mutations appear to act as loss-of-function mutations leading to an autosomal recessive form of the disease^6^. The physiological function of DJ-1 has not been fully elucidated. DJ-1 has been shown to be a multifunctional protein that participates in oxidative stress^7–11^, protein folding, glucose level, fertility, transcription and neuronal protection^5,12,13^. Discovery of the exact molecular mechanisms underlying disease due to DJ-1 loss-of-function mutations have been hampered in the past by the lack of valid small animal models. While the absence of nigral degeneration has been reported in DJ-1 knockout (KO) mice^14^, DJ-1 KO rats exhibit progressive nigral neurodegeneration with approximately 50% dopaminergic cell loss at 8 months of age^15^. Additionally, DJ-1 KO rats display significant motor impairments starting at 4 months^15^.

In both familial and sporadic PD, defects in mitochondria have been well-documented, and mitochondrial alterations have been reported in animal models of PD^16–18^. In the brain, synaptic mitochondria may be exceptionally vulnerable to damage, due to the high energetic demands at the synapse and the distance from the soma and nucleus for repair and/or replacement. The goal of this study was to assess whether the loss of DJ-1 in this rat model would result in noticeable changes in mitochondria at the striatal synapse, a key site of nigrostriatal neurotransmitter action. Therefore, we studied the proteomic and bioenergetic properties of striatal synaptic mitochondria from young adult rats, before the onset of neuronal loss due to deficiency in DJ-1.

## Results

### Proteomic alterations in striatal synaptic mitochondria from DJ-1 deficient rats

Since PD-related alterations in the nigrostrial pathway involve the loss of dopaminergic neuronal cell bodies in the SNpc and their associated nerve terminals in the striatum, we hypothesized that striatal synaptic mitochondria may be affected early in the disease process. We first examined the protein composition of these mitochondria, quantifying the striatal synaptic mitochondrial proteome in male wild-type (WT) and DJ-1 KO rats at 3 months, an age prior to reported motor deficits that start at 4 months, and significant neuronal loss observed at 8 months^15^. Striatal synaptic mitochondria were isolated and lysed, the proteins enzymatically digested, and the resultant peptides analyzed by sequential window acquisition of all theoretical fragment-ion spectra mass spectrometry (SWATH-MS), a data-independent acquisition (DIA) technique that using prior knowledge about the chromatographic and mass spectrometric behavior of peptides of interest in the form of spectral libraries, systematically in an unbiased fashion fragments all ionized peptides of a given sample that fall within a specified mass range using large precursor isolation windows^15^.

**Figure 1:**
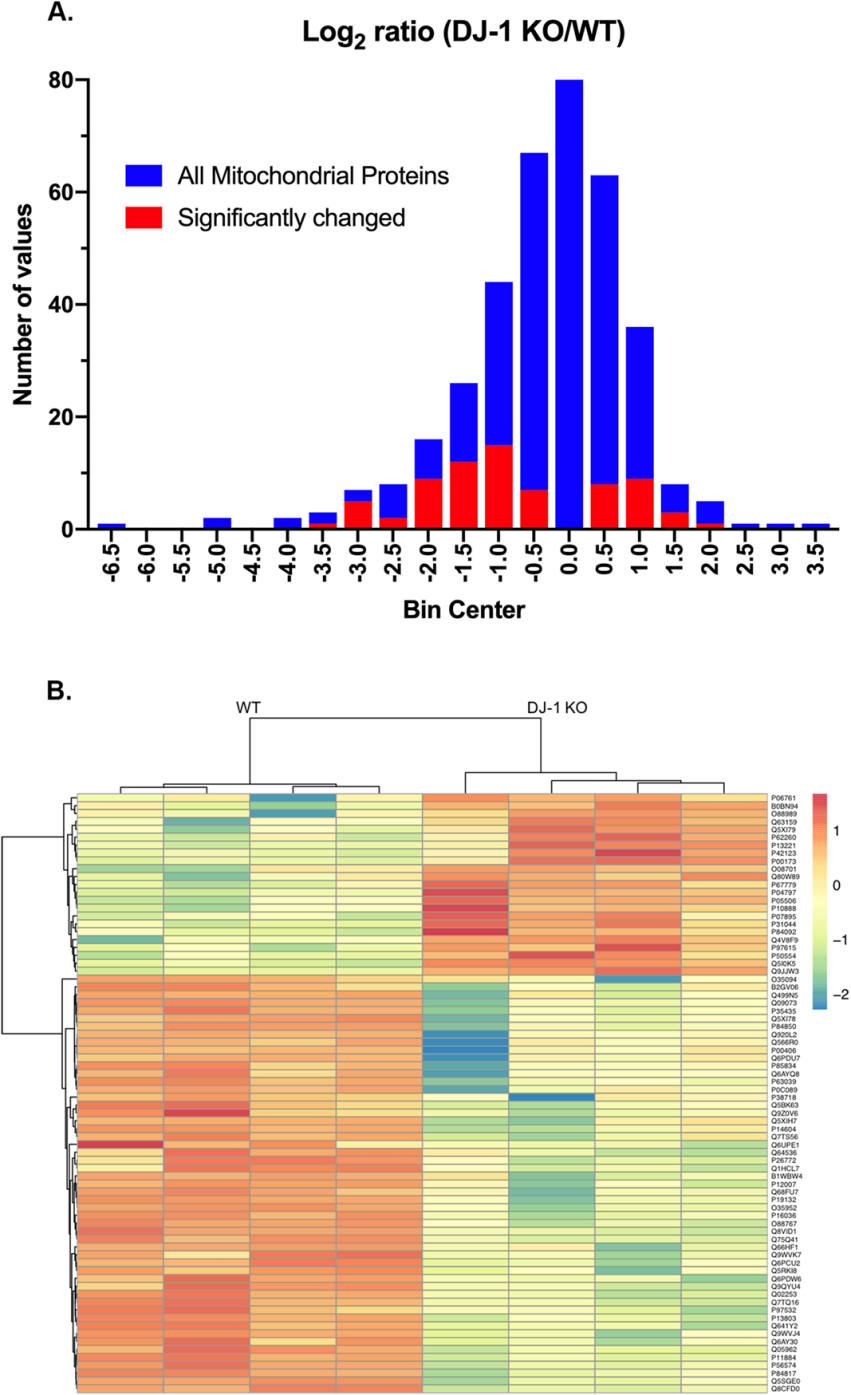
Differentially expressed mitochondrial proteins in striatal synaptic mitochondria from DJ-1 KO rats using SWATH-MS. (**A**) Histogram ratios (log_2_) of proteins in DJ-1 KO versus WT rats showing the distribution of all 371 mitochondrial proteins and the 76 differentially expressed mitochondrial proteins. (**B**) Heat map of the 76 differentially expressed proteins in each biological replicate (n = 4). Protein expression values are expressed in log_10_ for visualization purposes.

A total of 932 proteins were quantified by SWATH-MS in all striatal synaptic mitochondrial samples from the 3-month-old WT and DJ-1 KO rats (Table S1). Of these 932 proteins, 371 were annotated as mitochondrial by the DAVID Bioinformatics Resources 6.8 database, and these 371 mitochondrial proteins were chosen for further analysis. The complete list of the 371 mitochondrial proteins and the quantification values for each biological replicate is provided in Table S2. It is worth noting that despite the stop codon introduced into the *Park7* gene, Park7 (DJ-1) protein could still be detected in the DJ-1 KO rats, but at a level of 6.77-fold less than in WT (log_2_ DJ-1 KO/WT = − 2.76). This is consistent with others’ findings in these DJ-1 KO rats by both proteomics^19^ and Western blotting^15^. In addition, only peptides proximal to the mutation introduced in DJ-1 to yield the DJ-1 KO rats were found by mass spectroscopy in the DJ-1 KO rats, whereas a peptide distal to the mutation could also be found in WT rats, supporting the truncation, and likely nonsense mediated decay of the mutant protein in the DJ-1 KO rats. To assess the reproducibility of SWATH-MS among the four biological replicates in each rat strain, correlation analysis was performed and the Pearson’s correlation coefficient (r^2^) was 0.90 to 0.95 for WT and 0.83 to 0.93 for DJ-1 KO (Supplemental Fig. 2), suggesting high reproducibility of the quantitative SWATH-MS data between biological replicates. Using a false discovery rate < 0.05 for thresholds of differential expression, 76 differentially expressed mitochondrial proteins were found in DJ-1 KO rats as compared to their WT counterparts (Table S3). The log_2_ DJ-1 KO/WT ratio of all 371 mitochondrial proteins as well as the 76 differentially expressed mitochondrial proteins is graphed in Fig. 1A. Hierarchical clustering of the expression values (log_10_ DJ-1 KO/WT) for the 76 differentially expressed mitochondrial proteins revealed that around 70% were down-regulated and 30% were upregulated in DJ-1 KO rats (Fig. 1B).

**Figure 2:**
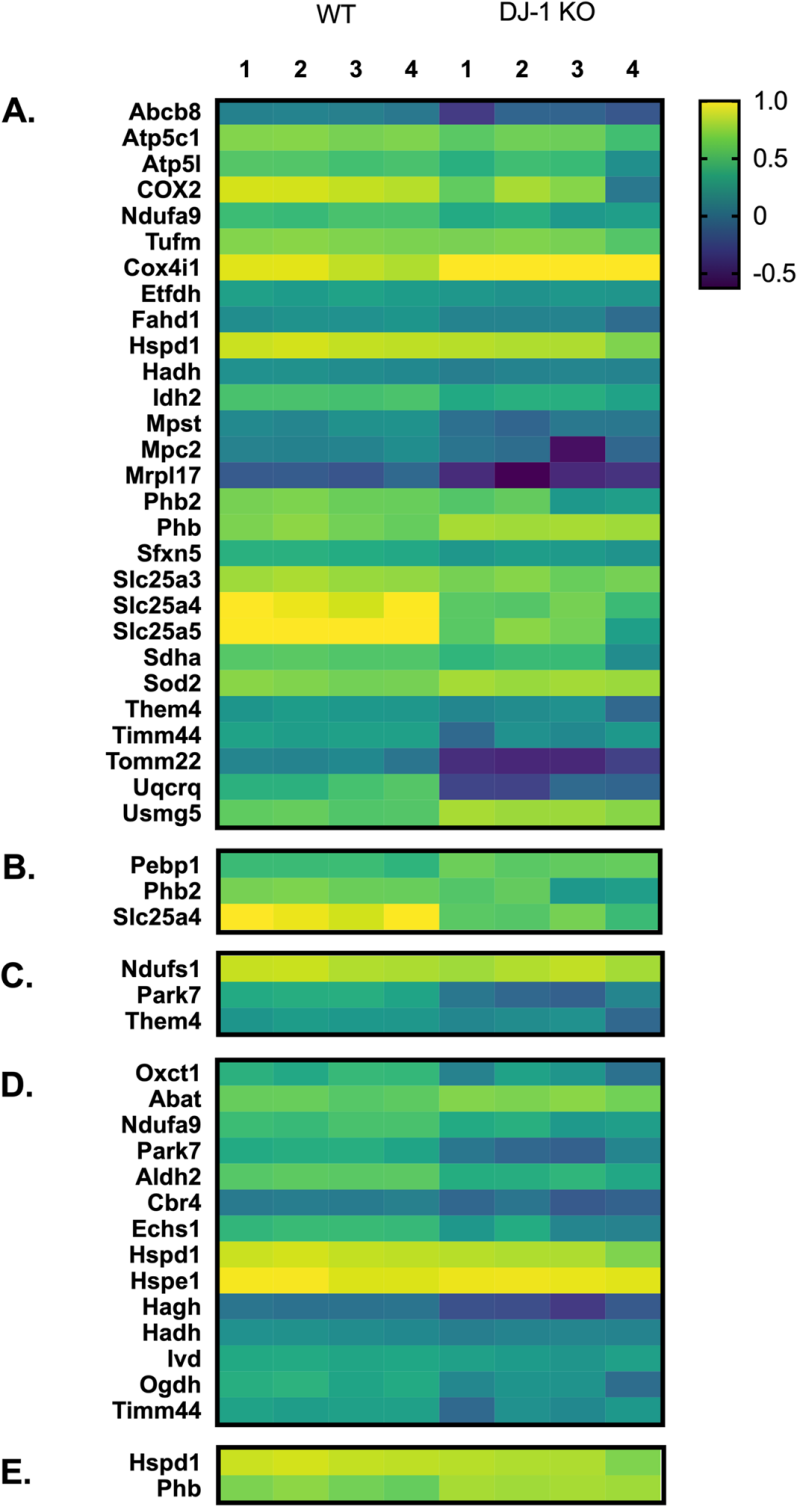
Mitochondrial localization of the differentially expressed proteins in the striatal synaptic mitochondria from DJ-1 KO rats. Heat maps depicting the expression levels for proteins differentially expressed in DJ-1 KO rats based on their predicted mitochondrial localization: (**A**) mitochondrial inner membrane (**B**) mitochondrial outer membrane (**C**) mitochondrial intermembrane space (**D**) matrix (**E**) crista. n = 4 biological replicates for each strain. Protein expression values are expressed in log_10_. The threshold was set so that the expression of truncated Park7 (DJ-1) in the DJ-1 KO rats was set to zero.

Since we analyzed proteins annotated to be mitochondrial as previously described, we examined where in the mitochondria they are predicted to localize (Fig. 2). In general, there was no localization bias, and they approximated the known representation of proteins in different mitochondrial compartments. Of the 371 quantified and 76 differentially expressed mitochondrial proteins, 30% and 37% were inner mitochondrial membrane proteins, 19% and 18% were mitochondrial matrix proteins, 9% and 4% were outer mitochondrial membrane proteins, 5% and 4% were intermembrane space proteins, and 1% and 4% were proteins in the crista, respectively.

**Figure 3:**
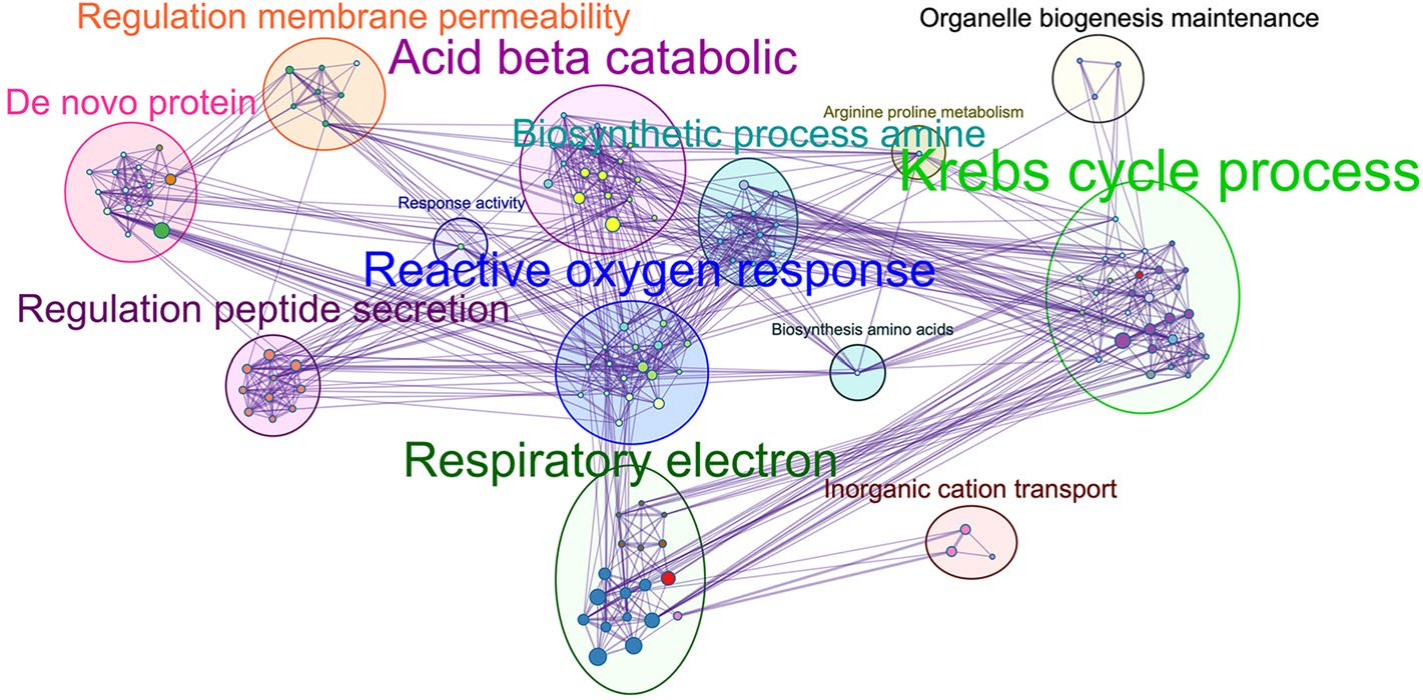
Overview of biological pathways altered in DJ-1 KO rats. Visualization (from Autoannotate) of biological processes predicted to be altered based on our proteomics data. Font size indicates the extent to which the pathways are affected by DJ-1 deficiency in striatal synaptic mitochondria (larger font = more affected).

Identification of the potential pathways afflicted by DJ-1 deletion in striatal synaptic mitochondria could improve our understanding of the disease caused by DJ-1 deficiency and potentially identify new targets. Using AutoAnnotate in Cytoscape, a bioinformatic pathway database, the top terms based on the list of 76 differentially expressed proteins in striatal synaptic mitochondria from DJ-1 KO rats were Krebs cycle process, reactive oxygen response, respiratory electron, and de novo protein synthesis. This revealed the cellular processes predicted to be altered by DJ-1 deficiency based on our proteomic results (Fig. 3).

**Figure 4:**
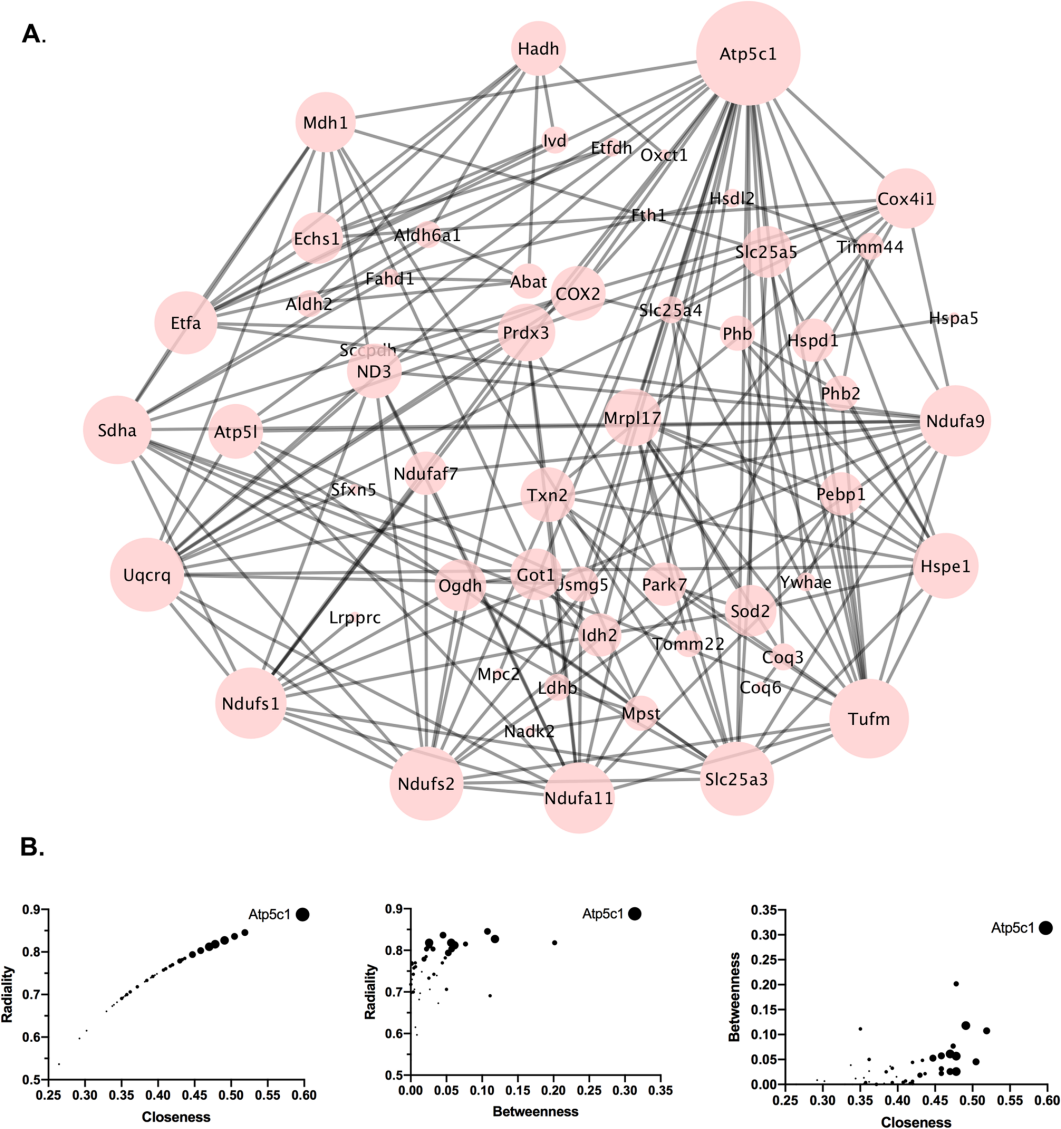
Protein–protein interactions (PPI) and centrality measures of differentially expressed synaptic mitochondrial proteins in DJ-1 KO vs WT rats. (**A**) PPI network of differentially expressed proteins centrality. Nodes degree was mapped based on the size, the larger the node the higher the degree. (**B**) Three centrality parameters (determined by CentiScaPe) were plotted against each other with the symbol size reflecting the degree of each differential expression (larger symbol = more affected).

Protein–protein interaction networks among the 76 differentially expressed mitochondrial proteins were investigated using STRING 11.0 (www.string-db.org). In STRING, the interactions were based on experiments, databases and co-expression with medium confidence greater than (0.400). STRING protein–protein interaction network information was exported to Cytoscape v. 3.7.2 and centrality analysis was performed. Centrality, a measure of the relative importance of a node (representing each) in the local network, was examined by determination of degree, betweenness, closeness, and radiality. In the network generated by STRING, centrality analysis was performed using the CentiScaPe 2.2 plug-in based on the degree of the node that represents the number of connections that each node has with other nodes, which gives a clear and simple overview of central nodes (Fig. 4A)^20^. The centrality analysis highlighted Atp5c1, a subunit of mitochondrial ATP synthase, the final enzyme in the electron transport chain (ETC), responsible for producing ATP from ADP and phosphate (Fig. 4B).

### Loss of DJ-1 alters striatal synaptic mitochondrial respiration in rats

**Figure 5:**
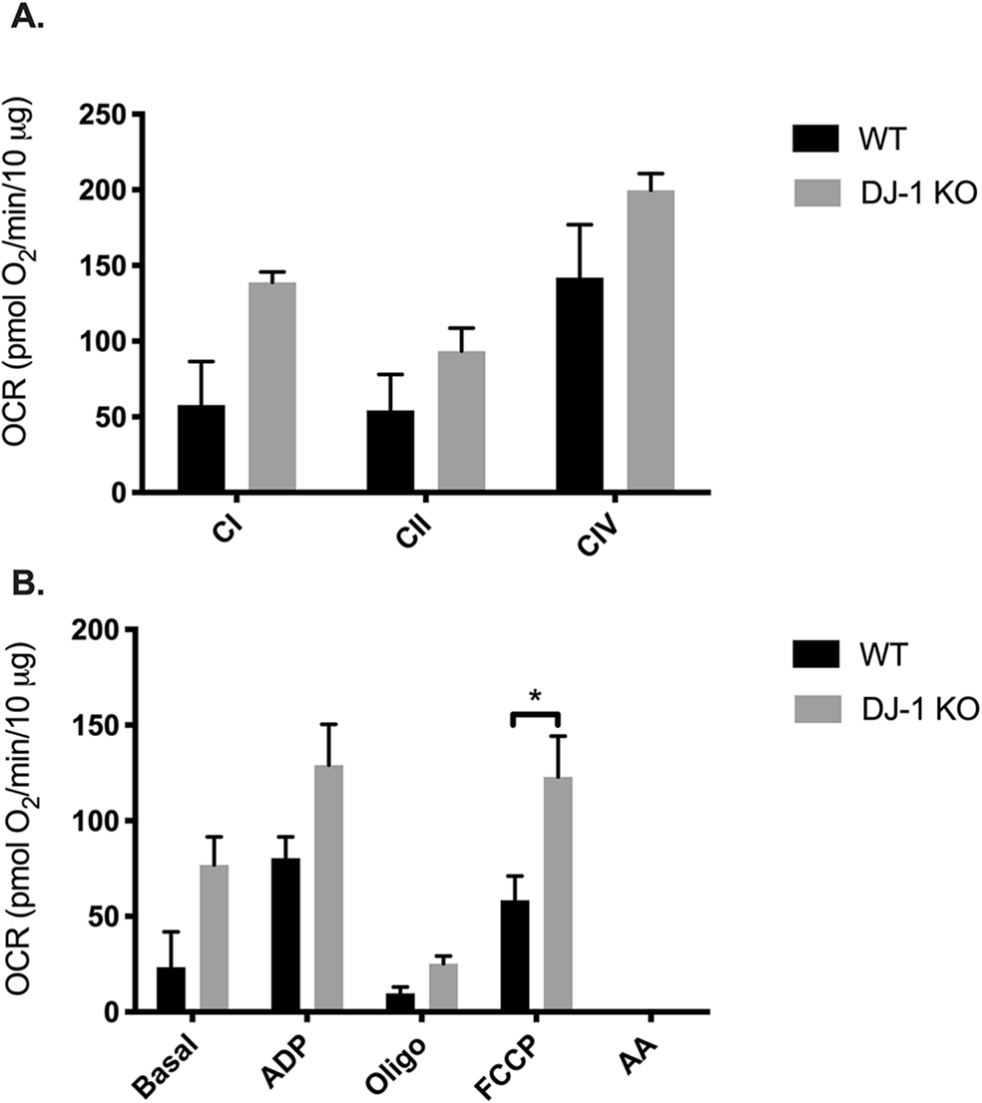
Bioenergetic analysis of striatal synaptic mitochondria from DJ-1 KO rats. Mitochondrial OCR was measured in striatal synaptic mitochondria isolated from 3-month-old WT and DJ-1 KO rats using a Seahorse XF24 analyzer (n = 3). (**A**) Electron flow assay assessing complex I- (pyruvate/malate), II- (succinate), and IV- (ascorbate/TMPD) driven respiration. Indicated are the mean ± SEM values. Two-way ANOVA revealed a significant effect of genotype (*p* < 0.0072). (**B**) Coupling assay assessing succinate-driven respiration. Mitochondria were administrated sequential injections of ADP, oligomycin, FCCP, and rotenone/antimycin A. Two-way ANOVA revealed a significant effect of genotype (*p* < 0.0003). Sidak’s post-hoc comparison test revealed FCCP, a depolarizing agent inducing maximal respiration, to be significantly increased in DJ-1 KO rats (indicated by *p < 0.05).

Functional assessment of the mitochondria was performed to investigate the impact of DJ-1 deficiency using the Seahorse XF24 analyzer. This is a standard technique to measure mitochondrial function by measuring the oxygen consumption rate (OCR) at baseline and after the subsequent injection of different ETC complex substrates and/or ETC complex inhibitors. First, the electron flow assay was used to examine sequential electron flow through different complexes of the ETC. Measurement of the OCR of DJ-1 KO synaptic mitochondria, compared to WT synaptic mitochondria, showed a genotype effect (*p* = 0.0072) of enhanced oxygen consumption in the DJ-1 deficient rats compared to WT, but did not show significant changes in the electron flow through different ETC complexes: complex I (*p* = 0.0741), complex II (*p* = 0.5617), and complex III (*p* = 0.2578) (Fig. 5A). Then, the coupling assay was used to examine the degree of coupling between the ETC and oxidative phosphorylation machinery (OXPHOS). Synaptic mitochondria from DJ-1 deficient rats similarly consumed more oxygen throughout the conditions assessed (two-way ANOVA, effect of genotype *p* < 0.0003), and for the individual conditions with the depolarizing agent FCCP, which induces maximal respiration, showing a significant increase (*p* < 0.05) in DJ-1 KO by Sidak’s post-hoc test). While basal OCR was higher in DJ-1 KO compared to WT it did not reach significance (*p* = 0.0506) nor did the OCR after ADP (*p* = 0.089), and oligomycin (*p* = 0.9354) treatment (Fig. 5B).

As mentioned above, the majority of the significantly altered proteins are located in the inner membrane of the mitochondria, where the ETC resides. Alteration of ETC complex I subunits represents 5.3% of the differentially expressed proteins, including Ndufs1, Ndufs2, Ndufa9, and Ndufa11. In addition, complexes II and III each have one, and complexes IV and V each two (1.3%, and 2.6%, respectively, with Atp5c1 being one of the complex V proteins) of the differentially altered proteins due to DJ-1 KO. Thus, the proteomic and functional studies suggest DJ-1 deficiency alters the ETC and oxidative phosphorylation in striatal synaptic mitochondria prior to nigral neurodegeneration in rats.

## Discussion

The selective, chronic, and progressive degeneration of dopaminergic neurons in the SNpc and the loss of dopaminergic nerve terminals in the striatum are responsible for motor deficits in PD, the most common neurodegenerative motor disorder in aging adults. Genetic mutations in several different genes have been discovered that lead to the development of hereditary PD, one of those genes is *Park7*, which encodes the protein DJ-1. Loss-of-function mutations in DJ-1 are linked to the early onset form of familial PD, but the pathological mechanism is still unknown^15^. In this study we investigated the proteomic and bioenergetic effects of loss of DJ-1 on striatal synaptic mitochondria prior to nigral neurodegeneration in rats. Previously, we reported proteomic alterations in non-synaptic mitochondria isolated from the striatum of male 3-month-old DJ-1 KO rats^21^. Although the proteomic analyses were performed by different methods, it was surprising that synaptic and non-synaptic mitochondria share only one differentially expressed protein, the complex III component Uqcrq. Unlike in synaptic mitochondria, non-synaptic mitochondria did not exhibit any alterations in complex I, II, and V subunit protein expression. The absence of proteomic alterations in non-synaptic mitochondria that are present in the synaptic mitochondria of DJ-1 KO rats might highlight the vulnerability of the synaptic mitochondrial population, and warrant further study. Of note, Seahorse functional analysis revealed an elevation in OCR in the non-synaptic mitochondria from the DJ-1 KO rats^21^, similar to our findings in the synaptic mitochondria studied here.

In examining these differentially expressed proteins, effects on the expression of multiple subunits of the ETC complexes stood out. Studies on mitochondria isolated from fibroblasts of patients with a DJ-1 mutation revealed reductions in Ndufs1, Ndufa9, Ndufb11, and Ndufb4, changes that could affect the ability to modulate the assembly or the stability of complex I^22^. In our study, similar to the findings in DJ-1 mutant PD fibroblasts, we identified a deficiency in Ndufs1 and Ndufa9 in DJ-1 KO rats. We also uncovered lower levels of the complex I subunit Ndufs2. Complex I deficiency has also been observed in neurons and peripheral tissues of PD patients^23–26^. These results suggest that complex I might have a central role in PD associated with DJ-1 deficiency.

**Figure 6:**
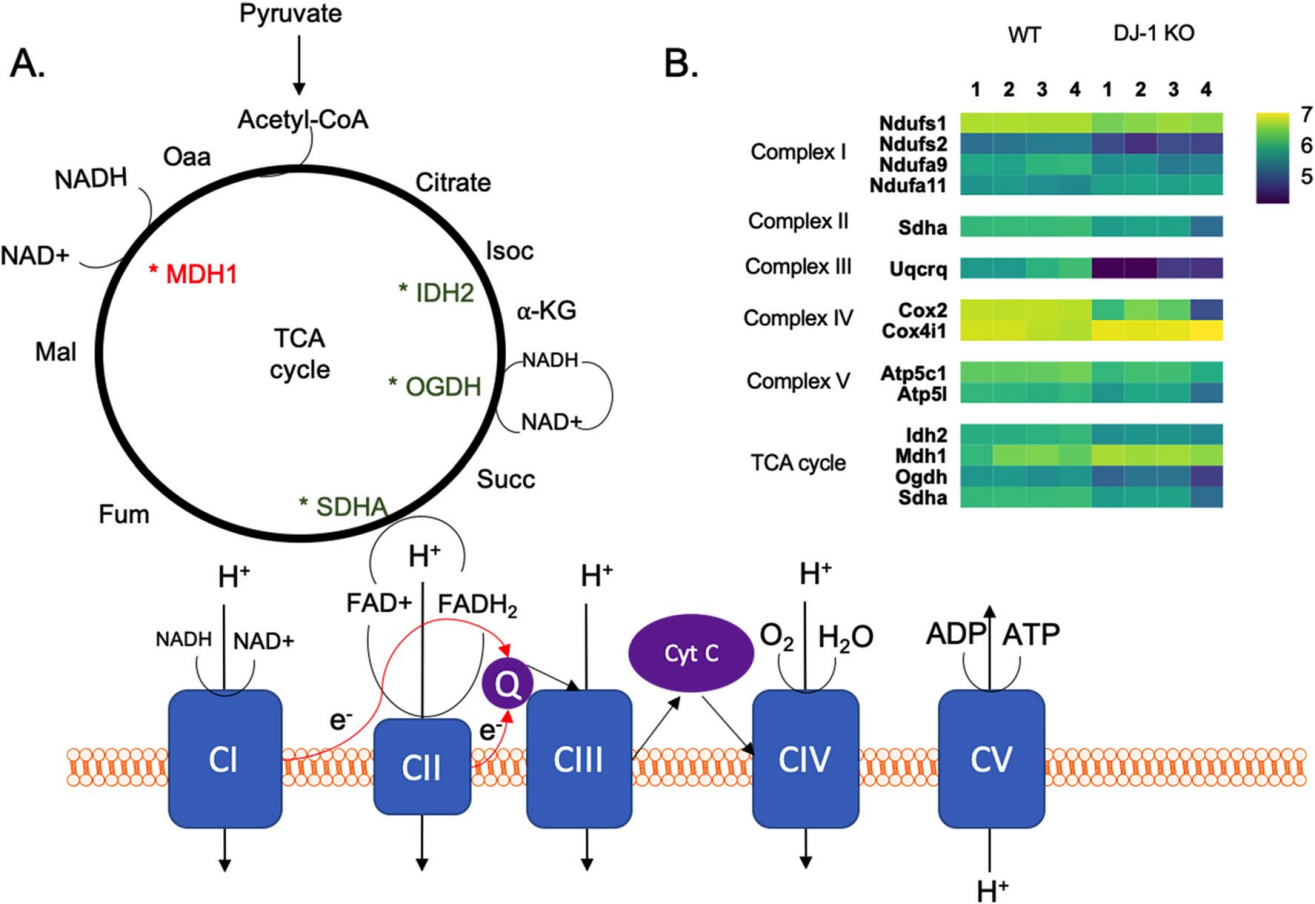
Quantitative analysis reveals changes in the expression of protein subunits in the ETC and TCA due to DJ-1 deficiency. (**A**) Schematic diagram of ETC and TCA cycle. (**B**) Heat map of ETC proteins and TCA cycle enzymes exhibiting differential expression in striatal synaptic mitochondria from DJ-1 KO compared to WT rats. Protein expression values are expressed in log_10_.

Mitochondrial complex II (also known as succinate dehydrogenase) has a dual function in the mitochondria. In the ETC it acts as an electron donor facilitating the conversion of ubiquinone to ubiquinol, and in the TCA cycle it catalyzed the oxidation of succinate into fumarate^27^. SNpc neurons from subjects with idiopathic PD were previously reported to manifest a significant reduction in Sdha, one of the four subunits of complex II^28^. Similarly, we found a down-regulation in Sdha in the DJ-1 KO rats, potentially linking proteomic changes in the TCA cycle to the ETC. Reductions in other enzymes involved in the TCA cycle were also found in DJ-1 KOs, isocitrate dehydrogenase 2 (Idh2) and oxoglutarate dehydrogenase (Ogdh), while one enzyme, malate dehydrogenase 1 (Mdh1) was increased (Fig. 6).

Reduction in the mitochondrial complex III subunit Uqcrq (which binds to ubiquinone with cytochrome b) was similar to an alteration that we previously found in non-synaptic mitochondria isolated from DJ-1 KO rats at the same age (3 months) as stated above^21^. Cytochrome c oxidase (complex IV) catalyzes the transfer of electrons from reduced cytochrome c to O_2_ to produce H_2_O^29^. Among the complex IV subunits, Cox2 and Cox4i1 were decreased in the DJ-1 KO rats. ATP synthase (also known as complex V) is responsible for ATP synthesis. Here, we found significant reduction in two of its subunits, Atp5c1 and Atp5I, in DJ-1 KO rats, with Atp5c1 highlighted in the centrality analysis. Interestingly, Atp5c1 has been recently identified in computational analyses as important in Parkinson’s as well as Alzheimer’s disease^30,31^. While alterations were found in additional mitochondrial proteins, our analyses indicated a distinct focus on the ETC.

We anticipated finding changes in mitochondrial energetic function based on these results. We found that striatal synaptic mitochondria from DJ-1 KO rats did not show any functional changes compared to WT rats using the electron flow assay. While surprising given the proteomic data, it is possible that compensatory changes took place, or that we examined a young age and defects had yet to manifest. However, when OCR was examined using the coupling assay, DJ-1 KO rat striatal synaptic mitochondria showed a significant increase in respiration, which was well-illustrated in the extreme condition of depolarization with FCCP. Indeed, this is similar to our findings in striatal non-synaptic mitochondria also isolated from 3-month-old DJ-1 KO rats, which showed a significant increase in OCR^21^. This is in contrast to the mouse model, where loss of DJ-1 did not result in changes in respiration of isolated cortical mitochondria at 3 months^32^, or changes in mitochondria isolated from brain and muscle at 4–6-months^33^. Of note, in contrast to these studies in mice, we specifically assessed synaptic mitochondria from the striatum, a portion of which would be derived from dopaminergic neurons in the nigra, regions known to degenerate in PD and DJ-1 KO rats, potentially explaining the presence of mitochondrial functional changes uncovered here. Further, DJ-1 KO mice do not exhibit nigral neurodegeneration. In general, mitochondria isolated from DJ-1 KO rats displayed an increase in OCR, which would be expected to contribute to oxidative stress caused by over production of reactive oxygen species (ROS). However, DJ-1 silencing was found to elevate the production of ROS in renal proximal tubules cells^34^.

In conclusion, DJ-1 mutations are associated with the early-onset form of PD. This study documents the importance of DJ-1 in the regulation of the striatal synaptic mitochondrial proteome and energetic profile. While 76 mitochondrial proteins were differentially expressed, proteins of the TCA cycle and ETC appeared particularly susceptible to loss of DJ-1. Functionally, in the striatum of DJ-1 KO rats, synaptic mitochondrial respiration was increased, particularly manifested in the condition of maximal energy generation. This work improves our understanding of DJ-1 in PD progression and may help in identifying new therapeutic targets.

## Materials and methods

### Animals

DJ-1 KO and the congenic wild-type (WT) Long Evans Hooded (LEH) rats were obtained from SAGE Labs (and now available from Envigo). Generation and characterization of the DJ-1 KO rats have been described previously^15^. Genotype was verified by Western blot (Supplemental Fig. 1). Rats were kept in a temperature-controlled environment with a 12-h light/dark cycle, in addition to free access to rat chow and water. Three-month-old male rats were used. All experimental procedures were approved by the UNMC Institutional Animal Care and Use Committee. For animal experiments the “Guide For The Care And Use Of Laboratory Animals, 8th edition” (National Research Council of the National Academies), Public Health Service Policy on Humane Care and Use of Laboratory Animals (U.S. Department of Health and Human Services, National Institutes of Health, Office of Laboratory Animal Welfare), and UNMC policies were all followed.

### Brain dissection and synaptic mitochondrial isolation

Synaptic mitochondria were isolated as previously described^35,36^. All mitochondrial isolation steps were performed on ice or at 4 °C. Briefly, rats were anesthetized by isoflurane and sacrificed by decapitation. Right after euthanasia, brains were removed and placed on a pre-chilled petri dish. The petri dish was placed under a dissection microscope to dissect the striatum from the brain. The striatum was then transferred to a microcentrifuge tube containing ice-cold mitochondrial isolation media (MSHE + BSA) containing mannitol (210 mM), sucrose (70 mM), HEPES (5 mM), EGTA (1 mM), and fatty acid free (FAF)-BSA (0.5% w/v) (PH 7.2) to prepare the striatum for homogenization. The striatum was then homogenized (10 strokes) using a Dounce homogenizer containing ice-cold MSHE + BSA buffer. The homogenate was then centrifuged at 1300×*g* for 3 min; the supernatant was collected, and the pellet was suspended again in MSHE + BSA and recentrifuged at 1300×*g* for 3 min. Both supernatants were pooled together and centrifuged at 21,000×*g* for 10 min. The resulting supernatant was discarded, and the pellet was resuspended in Percoll to a final concentration of 15% Percoll, and layered on the top of 24% and 40% Percoll (100% Percoll was used to make the different components of the gradient, which contains MSHE + BSA (as above). Ultracentrifugation was performed for 8 min at 30,700×*g*. Synaptosomes (pinched off nerve terminals containing the presynaptic component including mitochondria) band between the 15% and 24% layers. These were collected and diluted in MSHE + BSA.

To isolate synaptic mitochondria, the synaptosomal fraction was transferred to a nitrogen cavitation vessel (Parr Instrument Company, Moline, IL). The pressure in the vessel was calibrated to 900 psi for 15 min followed by depressurization to atmospheric pressure. The resulting material were then added on top of 24% Percoll and ultracentrifuge at 30,700×*g* for 10 min. After discarding the supernatant, the pellet containing synaptic mitochondria was resuspended in MSHE + BSA and recentrifuged at 8000×*g* for 10 min. Mitochondrial assay solution (MAS, 1x: 70 mM sucrose, 220 mM mannitol, 10 mM KH_2_PO_4_, 5 mM MgCl_2_, 2 mM HEPES, 1 mM EGTA, and 0.2% (w/v) FAF-BSA PH 7.2) was used to wash the pellets twice. Pellets then were resuspended in a minimal volume of MAS. Total concentrations of mitochondria were determined using the Pierce BCA Protein Assay kit with albumin standards (Thermo Fischer Scientific, Waltham, MA). Finally, the isolated mitochondria were either lysed and frozen for proteomic studies or immediately used for bioenergetic analysis.

### Sample preparation for SWATH-MS proteomics

Synaptic mitochondrial lysates isolated from rat striatum were prepared for analysis by digestion with trypsin (Promega, Madison, WI) using filter-aided sample preparation (FASP) on a 20 μm filter (Pall Corporation, Ann Arbor, MI)^8^. Peptides were then desalted using Oasis mixed-mode weak cation exchange cartridges (Waters, Milford, MA), then dehydrated with a Savant ISS 110 SpeedVac concentrator (Thermo Fischer Scientific, Waltham, MA). The dried pellets were dissolved in 0.1% formic acid (FA) (Fischer Scientific, Hampton, NH) and quantified using the Scopes method^37^ on a NanoDrop 2000 UV–vis Spectrophotometer (Thermo Fischer Scientific, Waltham, MA) at 205 nm absorbance.

### SWATH data acquisition

Sample peptides from the striatal synaptic mitochondrial lysates from LEH and DJ-1 KO rats were analyzed in quadruplicate by nano-LC–MS /MS in SWATH-MS mode on a 5600 TripleTOF instruments (SCIEX) and targeted data extraction was performed as previously described^21,38^. The spectral library was generated using ProteinPilot (Version 4.5, SCIEX) where the search was performed against *Rattus norvegicus* UniProt proteome UP000002494 that contain 8023 Swiss-Prot reviewed proteins. Then, every fragment ion chromatogram was extracted and integrated automatically with PeakView (Version 2.1, SCIEX). MarkerView (Version 1.2.1) was used for data normalization to median peak ratios.

### Bioinformatic analysis

The Database for the Annotation, Visualization and Integrated Discovery (DAVID, https://david.abcc.ncifcrf.gov) Bioinformatics Resources 6.7 was used for functional annotation^39^. Gene Ontology (GO) was generated by STRING version 11.0 (https://string-db.org)^40^. Cytoscape (https://cytoscape.org), an open-source software that is used to generate a molecular interaction network^41^. Autoannotate (https://autoannotate.readthedocs.io/en/latest/) was used to generate an overview of the cellular pathways and processes affected in DJ-1 KO rats^42^.

### Mitochondrial bioenergetics assays

Oxygen consumption rates (OCR) for the isolated striatal mitochondria were measured using the Seahorse XFe24 Extracellular Flux Analyzer (Seahorse Bioscience) electron flow and coupling assays, which were described previously^35,43^. Each independent biological replicate (n = 3 for LEH and DJ-1 KO) was measured using 3–4 technical replicate wells. In the 24-well Seahorse plate, Isolated synaptic mitochondria were plated in 20 μl MAS with the proper substrates and inhibitors for Flux assay (complex I, 10 mM pyruvate and 2 mM malate as substrate; complex II, 10 mM succinate as substrate in the presence of 2 μM rotenone (Rot); complex IV, 10 mM ascorbate with 100 μM tetramethylphenylenediamine (TMPD) as substrate in the presence of 4 μM antimycin A (AA). Data were then normalized by Rot or AA. In the coupling assay of the mitochondria, 1 ml MAS (with or without inhibitors or substrates) was added to each well and incubated at 37 °C to equilibrate temperature. Sequential injections’ final concentrations were 4 mM ADP, 2.5 μM oligomycin (Oligo), 4 μM carbonylcyanide-*p*-triflouromethoxyphenylhydrazone (FCCP), and 2 μM Rot 4 μM AA. Data were then normalized by AA.

### Statistical analysis

Statistical analysis and heat maps of proteins expressions values were generated with Prism (version 7.00 for Windows, GraphPad Software, La Jolla California USA). Briefly, DAVID was used to identify the mitochondrial proteins. The mean and the SD of all four biological replicates was used to generate a plot. Then, multiple t test correction was done using two-stage set-up method of Benjamini, Krieger and Yekutieli method. Proteins with q < 0.05 were deemed differentially expressed. Each row was analyzed individually, without assuming a consistent SD.

The Seahorse Wave 2.2.0 software package was used for calculating the data and Prism (GraphPad Software) was used for graph generation. For the statistics, statistical analysis was conducted in Prism using two-way ANOVA between groups corrected by the Sidak method.

## Supplementary information

Supplementary Information 1.

Supplementary Information 2.

## References

[1] Jankovic J (2008). Parkinson's disease: clinical features and diagnosis. J Neurol Neurosurg Psychiatry.

[2] Klein C, Westenberger A (2012). Genetics of Parkinson's disease. Cold Spring Harb Perspect Med.

[3] Zhang L (2005). Mitochondrial localization of the Parkinson's disease related protein DJ-1: implications for pathogenesis. Hum Mol Genet.

[4] Nagakubo D (1997). DJ-1, a novel oncogene which transforms mouse NIH3T3 cells in cooperation with ras. Biochem Biophys Res Commun.

[5] Bonifati V (2003). Mutations in the DJ-1 gene associated with autosomal recessive early-onset parkinsonism. Science.

[6] Pankratz N (2006). Mutations in DJ-1 are rare in familial Parkinson disease. Neurosci Lett.

[7] Yanagida T (2009). Oxidative stress induction of DJ-1 protein in reactive astrocytes scavenges free radicals and reduces cell injury. Oxid Med Cell Longev.

[8] Mitsumoto A (2001). Oxidized forms of peroxiredoxins and DJ-1 on two-dimensional gels increased in response to sublethal levels of paraquat. Free Radic Res.

[9] Yokota T (2003). Down regulation of DJ-1 enhances cell death by oxidative stress, ER stress, and proteasome inhibition. Biochem Biophys Res Commun.

[10] Canet-Aviles RM (2004). The Parkinson's disease protein DJ-1 is neuroprotective due to cysteine-sulfinic acid-driven mitochondrial localization. Proc Natl Acad Sci U S A.

[11] Kinumi T, Kimata J, Taira T, Ariga H, Niki E (2004). Cysteine-106 of DJ-1 is the most sensitive cysteine residue to hydrogen peroxide-mediated oxidation in vivo in human umbilical vein endothelial cells. Biochem Biophys Res Commun.

[12] Cookson MR (2012). Parkinsonism due to mutations in PINK1, parkin, and DJ-1 and oxidative stress and mitochondrial pathways. Cold Spring Harb Perspect Med.

[13] Dias V, Junn E, Mouradian MM (2013). The role of oxidative stress in Parkinson's disease. J Parkinsons Dis.

[14] Chandran JS (2008). Progressive behavioral deficits in DJ-1-deficient mice are associated with normal nigrostriatal function. Neurobiol Dis.

[15] Dave KD (2014). Phenotypic characterization of recessive gene knockout rat models of Parkinson's disease. Neurobiol Dis.

[16] Grunewald A, Kumar KR, Sue CM (2018). New insights into the complex role of mitochondria in Parkinson's disease. Prog Neurobiol.

[17] Helley MP, Pinnell J, Sportelli C, Tieu K (2017). Mitochondria: A Common Target for Genetic Mutations and Environmental Toxicants in Parkinson's Disease. Front Genet.

[18] Bose A, Beal MF (2016). Mitochondrial dysfunction in Parkinson's disease. J Neurochem.

[19] Hauser DN (2017). Hexokinases link DJ-1 to the PINK1/parkin pathway. Mol Neurodegener.

[20] Scardoni G (2014). Biological network analysis with CentiScaPe: centralities and experimental dataset integration. F1000Res.

[21] Stauch KL (2016). SWATH-MS proteome profiling data comparison of DJ-1, Parkin, and PINK1 knockout rat striatal mitochondria. Data Brief.

[22] Di Nottia M (2017). DJ-1 modulates mitochondrial response to oxidative stress: clues from a novel diagnosis of PARK7. Clin Genet.

[23] Schapira AH (1989). Mitochondrial complex I deficiency in Parkinson's disease. Lancet.

[24] Benecke R, Strumper P, Weiss H (1993). Electron transfer complexes I and IV of platelets are abnormal in Parkinson's disease but normal in Parkinson-plus syndromes. Brain.

[25] Parker WD, Swerdlow RH (1998). Mitochondrial dysfunction in idiopathic Parkinson disease. Am J Hum Genet.

[26] Ambrosi G (1842). Bioenergetic and proteolytic defects in fibroblasts from patients with sporadic Parkinson's disease. Biochim Biophys Acta.

[27] Stepanova A, Shurubor Y, Valsecchi F, Manfredi G, Galkin A (1857). Differential susceptibility of mitochondrial complex II to inhibition by oxaloacetate in brain and heart. Biochim Biophys Acta.

[28] Grunewald A (2016). Mitochondrial DNA depletion in respiratory chain-deficient parkinson disease neurons. Ann Neurol.

[29] Welchen E, Garcia L, Mansilla N, Gonzalez DH (2014). Coordination of plant mitochondrial biogenesis: keeping pace with cellular requirements. Front Plant Sci.

[30] Prajapati R, Emerson IA (2020). Gene prioritization in Parkinson's disease using human protein-protein interaction network. J Comput Biol.

[31] Tao Y (2020). The predicted key molecules, functions, and pathways that bridge mild cognitive impairment (MCI) and Alzheimer's disease (AD). Front Neurol.

[32] Giaime E, Yamaguchi H, Gautier CA, Kitada T, Shen J (2012). Loss of DJ-1 does not affect mitochondrial respiration but increases ROS production and mitochondrial permeability transition pore opening. PLoS ONE.

[33] Hao LY, Giasson BI, Bonini NM (2010). DJ-1 is critical for mitochondrial function and rescues PINK1 loss of function. Proc Natl Acad Sci U S A.

[34] Cuevas S (2012). Role of renal DJ-1 in the pathogenesis of hypertension associated with increased reactive oxygen species production. Hypertension.

[35] Stauch KL, Purnell PR, Fox HS (2014). Quantitative proteomics of synaptic and nonsynaptic mitochondria: insights for synaptic mitochondrial vulnerability. J Proteome Res.

[36] Stauch KL, Purnell PR, Fox HS (2014). Aging synaptic mitochondria exhibit dynamic proteomic changes while maintaining bioenergetic function. Aging (Albany NY).

[37] Scopes RK (1974). Measurement of protein by spectrophotometry at 205 nm. Anal Biochem.

[38] Stauch KL (2016). Loss of Pink1 modulates synaptic mitochondrial bioenergetics in the rat striatum prior to motor symptoms: concomitant complex I respiratory defects and increased complex II-mediated respiration. Proteomics Clin Appl.

[39] da Huang W, Sherman BT, Lempicki RA (2009). Systematic and integrative analysis of large gene lists using DAVID bioinformatics resources. Nat Protoc.

[40] Szklarczyk D (2019). STRING v11: protein-protein association networks with increased coverage, supporting functional discovery in genome-wide experimental datasets. Nucleic Acids Res.

[41] Shannon P (2003). Cytoscape: a software environment for integrated models of biomolecular interaction networks. Genome Res.

[42] Kucera M, Isserlin R, Arkhangorodsky A, Bader GD (2016). AutoAnnotate: a cytoscape app for summarizing networks with semantic annotations. F1000Res.

[43] Villeneuve LM, Purnell PR, Boska MD, Fox HS (2016). Early expression of Parkinson's Disease-related mitochondrial abnormalities in PINK1 knockout rats. Mol Neurobiol.

