# Supplementary material for: Deletion of DJ-1 in rats affects protein abundance and mitochondrial function at the synapse
Source: Sci Rep. 2020 Aug 13;10:13719. doi: 10.1038/s41598-020-70486-0 (PMC7426919; doi:10.1038/s41598-020-70486-0)
Supplement: Supplementary file 1 — Supplementary Information 1. [file 41598_2020_70486_MOESM1_ESM.pdf]

Supplemental Figures for:

Deletion Of DJ-1 in Rats Affects Protein Abundance and Mitochondrial Function at the Synapse

Mohannad A. Almikhlafi[1,2], Kelly L. Stauch[1,3], Lance M. Villeneuve[3], Phillip R. Purnell[3], Benjamin G. Lamberty[1,3], Howard S. Fox[1,3*]

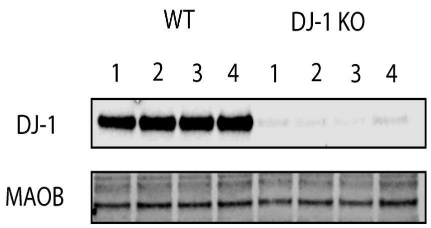

Supplemental Figure 1A. DJ-1 protein expression.

Western blot confirmation of lack of expression of DJ-1 in striatal mitochondria from DJ-1 KO rats, compared to wild-type rats. The mitochondrial protein monoamine oxidase B (MAOB) was used as a loading control.

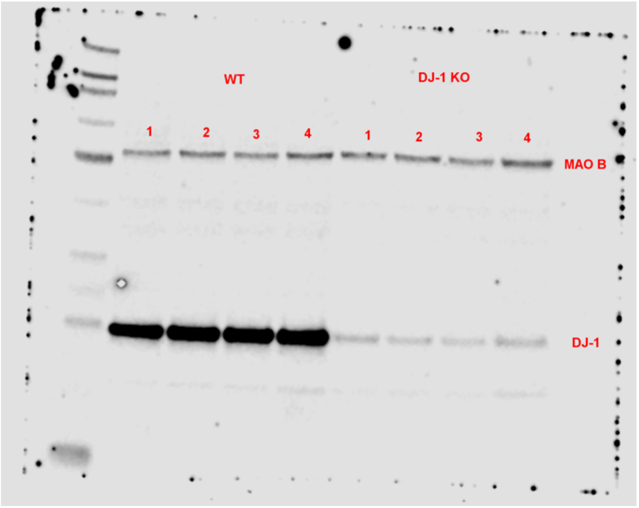

Supplemental Figure 1B. Full uncropped Western blot supporting Supplemental Figure 1A. Molecular mass markers are on far left, followed by protein 4 independent biological replicate wild-type rats, and 4 independent biological replicate DJ-1 KO rats, probed with antibodies to DJ-1 and MAOB.

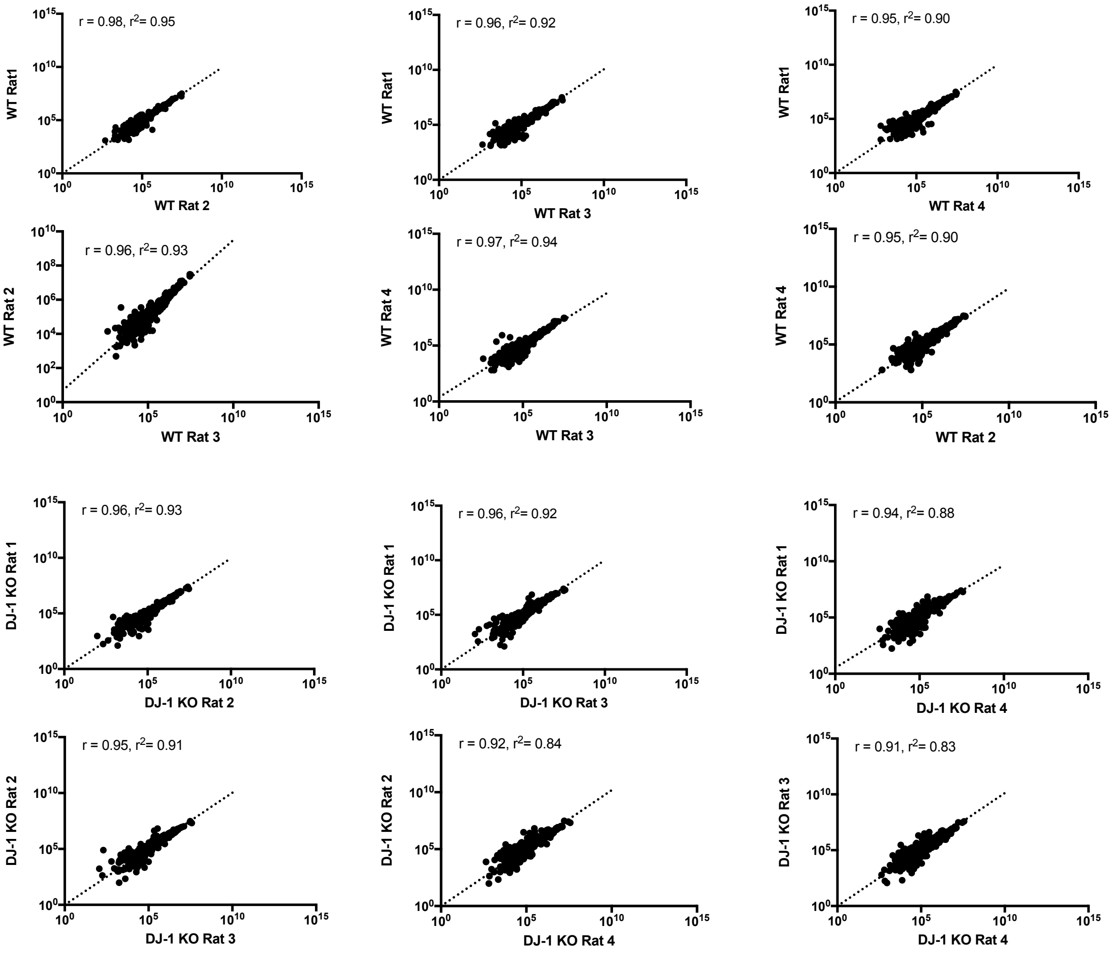

Supplemental Figure 2. Reproducibility of SWATH-MS proteomics between biological replicates of WT and DJ-1 KO synaptic mitochondria.

Scatter plot of the SWATH-MS intensity values determined in one biological replicate compared to another biological replicate for all 932 quantified proteins.
